# Septal Ablation Versus Surgical Myomectomy for Hypertrophic Obstructive Cardiomyopathy

**DOI:** 10.1007/s11886-021-01600-5

**Published:** 2021-10-01

**Authors:** F. Pelliccia, H. Seggewiss, F. Cecchi, P. Calabrò, G. Limongelli, O. Alfieri, P. Ferrazzi, M. H. Yacoub, I. Olivotto

**Affiliations:** 1 Department of Cardiovascular Sciences, University Sapienza, Via del Policlinico 155, 00161 Rome, Italy; 2 Comprehensive Heart Failure Center (CHFC), Deutsches Zentrum Für Herzinsuffizienz (DZHI), Universitätsklinikum Würzburg, Würzburg, Germany; 3 Department of Cardiovascular, Neural and Metabolic Sciences, IRCCS Istituto Auxologico Italiano, San Luca Hospital, Milan, Italy; 4 Department of Translational Medical Sciences, University of Campania ‘Luigi Vanvitelli’, Naples, Italy; 5 Institute of Cardiovascular Sciences, University College of London, St. Bartholomew’s Hospital, London, UK; 6 Department of Cardiovascular and Thoracic Surgery, San Raffaele University Hospital, Milan, Italy; 7 Hypertrophic Cardiomyopathy Center, Policlinico Di Monza, Monza, Italy; 8 Heart Science Centre, National Heart and Lung Institute, Imperial College London, London, UK; 9 Cardiomyopathy Unit, Careggi University Hospital, Florence, Italy

**Keywords:** Alcohol septal ablation, Hypertrophic cardiomyopathy, Gradient, Left ventricular outflow tract, Myectomy, Obstruction

## Abstract

**Purpose of Review:**

Patients with hypertrophic cardiomyopathy (HCM) who have left ventricular outflow tract obstruction (LVOTO) often experience severe symptoms and functional limitation. Relief of LVOTO can be achieved by two invasive interventions, i.e., surgery myectomy and alcohol septal ablation (ASA), leading in experienced hands to a dramatic improvement in clinical status. Despite extensive research, however, the choice of the best option in individual patients remains challenging and poses numerous clinical dilemmas.

**Recent Findings:**

Invasive strategies have been recently incorporated in recommendations for the diagnosis and treatment of HCM on both sides of the Atlantic. These guidelines are based on a bulk of well-designed but retrospective studies as well as on expert opinions. Evidence now exists that adequate evaluation and management of HCM requires a multidisciplinary team capable of choosing the best available options.

**Summary:**

Management of LVOTO still varies largely based on local expertise and patient preference. Following the trend that has emerged for other cardiac diseases amenable to invasive interventions, the concept of a “HCM heart team” is coming of age.

## Introduction

Hypertrophic cardiomyopathy (HCM) is a complex cardiovascular disorder characterized by unexplained left ventricular (LV) thickening in the absence of hemodynamic overload due to cardiac or systemic disease [1]. Two-thirds of HCM patients suffer from the obstructive type of the condition, characterized by highly dynamic LV outflow tract obstruction (LVOTO) and variable manifestations in the form of dyspnea, angina pectoris, and presyncope or syncope. LVOTO is caused by systolic anterior movement (SAM) of anomalous mitral valve leaflets, contacting the septum at the subaortic level [2]. Relief of obstruction by pharmacological or invasive interventions usually leads to dramatic improvement in symptoms [3].

Pharmacologic therapy with betablockers, verapamil, and disopyramide represents the first-line strategy. However, when symptoms prove refractory to medical therapy and obstruction persists, invasive septal reduction strategies should be taken into consideration. The two established options currently available are surgical myectomy and alcohol septal ablation (ASA). Unfortunately, there are no randomized trials comparing myectomy and ASA and none are anticipated: a definitive comparison with regard to outcome is probably not feasible in a disease characterized by low event rates such as HCM. As a consequence, European and American scientific guidelines do not provide class I recommendations for any of these invasive options [4, 5] and the choice in the individual patient is largely determined by clinical judgement, local expertise, and patient preference. Following the trend that has emerged for other invasive cardiovascular treatments, adequate evaluation and management of obstructive HCM today requires a multidisciplinary team capable of choosing the best available options, in order to minimize risk of complications and warrant optimal immediate and long-term results [6••].

This review aims to provide an update of invasive options for the management of LVOT in HCM, highlighting areas for multidisciplinary integration and future development.

## Obstruction in HCM

The pathophysiology of HCM was first described by Braunwald et al. [7] and Wigle et al. [8] who emphasized the obstructive nature of the disease. LV outflow obstruction is typically dynamic and largely influenced by changes in LV loading and contractility. It leads to increased LV systolic pressure, which in turn gives rise to a complex interplay of elevated wall stress, prolongation of ventricular relaxation, abnormal LV filling, increased filling pressure, secondary mitral regurgitation, myocardial ischemia, and reduction in cardiac output.

Multiple mechanisms are involved in the pathophysiology of LVOTO in HCM. The anatomic determinant is represented by hypertrophied basal anteroseptal wall and redundant mitral leaflets, coupled with a small-sized ventricular cavity. The functional determinant of obstruction is the systolic anterior motion (SAM) of the mitral valve and the consequent mitral-septal contact [9]. SAM is mainly the consequence of primary abnormalities of the mitral apparatus (i.e., papillary muscle hypertrophy and displacement, leaflet elongation, and changes in chordal attachments). In addition, evidence now exists that altered flow vectors generated in the LV cavity along with changes in outflow tract geometry favor the contact between the anterior leaflet of the mitral valve and the hypertrophied septum [10]. These synergistic mechanisms push the leaflets into the outflow tract (“drag forces”) resulting in LVOTO and eccentric mitral regurgitation [11]. Abnormally positioned papillary muscles and abnormal papillary attachment to the leaflets contribute to LVOTO.

LVOTO should be routinely sought during routine evaluation of HCM. Around 70% of patients exhibit significant obstruction with a peak pressure difference of more than 30 mm Hg [2]. In about 30–35%, significant obstruction is present at rest, while a gradient can be demonstrated only under exercise in the remaining patients. American guidelines advise exercise stress echocardiography for identification of latent obstruction in all patients with HCM [5]. Conversely, European recommendations warrant the search for latent LVOTO by exercise echocardiography in presence of symptoms (i.e., dyspnea, chest pain, exercise limitation, and/or impaired consciousness), but not in asymptomatic patients unless “relevant to lifestyle advice and decisions on medical treatment” [4].

Obstruction in HCM should always be properly diagnosed and adequately treated, as it is a major determinant of patients’ clinical and hemodynamic status, and a determinant of outcome. Unfortunately, a large proportion of patients with dynamic obstruction remain symptomatic despite optimal drug treatment. In such occurrence, invasive treatment of LVOTO should be considered in symptomatic patients with an LVOT gradient ≥ 50 mm Hg, in spite of maximally tolerated drug therapy. Over the past decades, surgical septal myectomy and ASA have become the two established options in this challenging subset.

## Surgical Myectomy

**Fig. 1:**
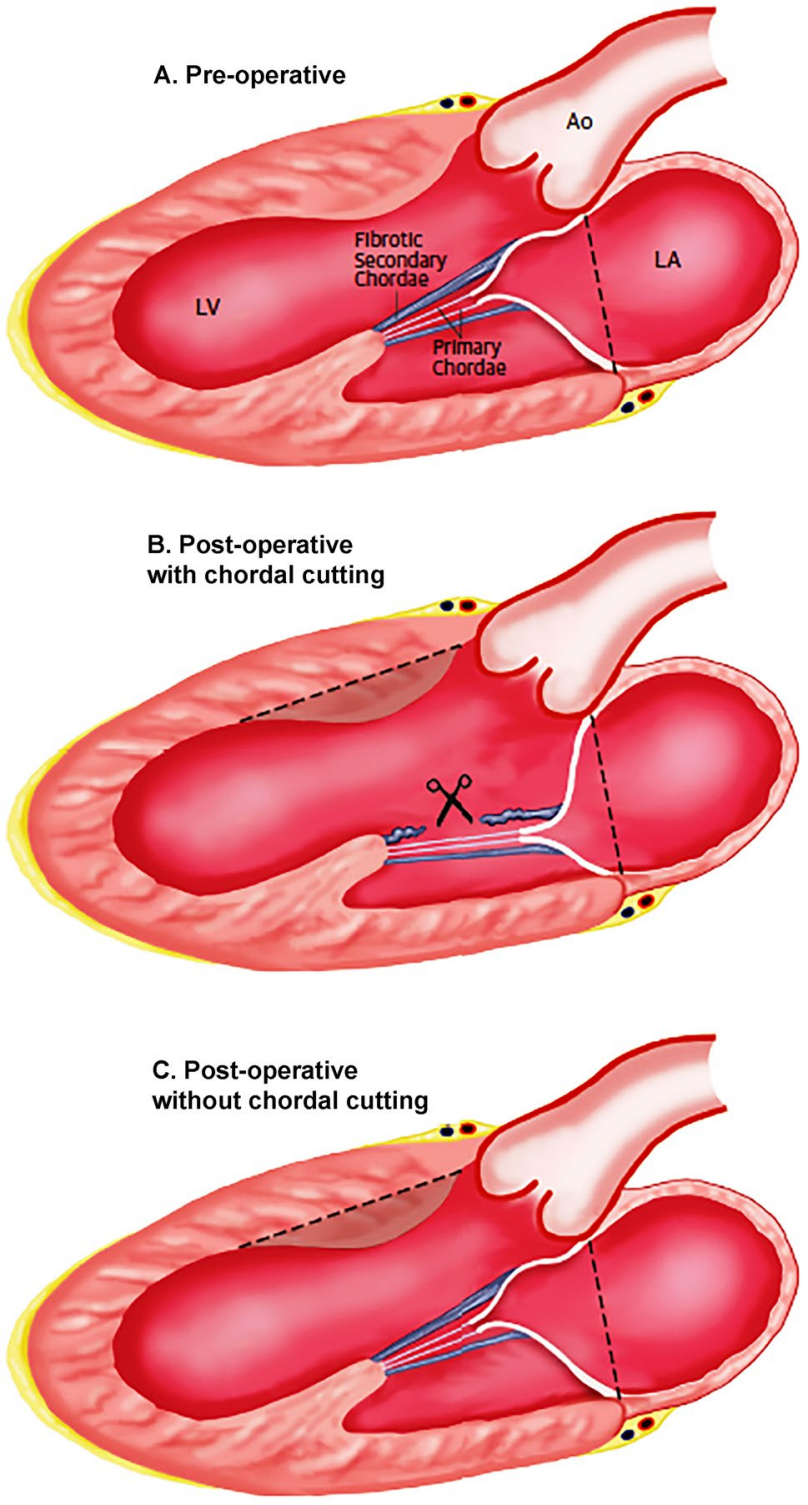
Secondary chordal cutting in obstructive HCM: effects of secondary chordal cutting on the geometry and function of the mitral valve apparatus. **A** In patients with obstructive hypertrophic cardiomyopathy, fibrotic and retracted mitral valve secondary chordae contribute to displace the body of the anterior leaflet into the left ventricular outflow tract. **B** Cutting selected abnormal chordae (in combination with a shallow septal myectomy) moves the mitral valve apparatus and leaflet coaptation point away from the outflow tract to a more posterior and normal position in the left ventricular cavity, substantially increasing outflow tract size and decreasing mitral valve tenting area. **C** Isolated septal myectomy (i.e., without associated chordal cutting) does not alter the anterior displacement of the mitral valve apparatus. Ao, aorta; LA, left atrium; LV, left ventricle. Dashed lines indicate the changes in LA and LV morphology obtained with operation. ( Reproduced from: Pelliccia F et al. Int J Cardiol. 2020;304:86–92,with permission) [6••]

The septal myectomy operation was initially proposed by Morrow with the aim to restore normal LV hemodynamics by surgically abolishing LVOTO [12]. For decades, this procedure has been considered the gold standard for relief of obstruction in severely symptomatic HCM patients. The classical surgical myectomy consists in the transaortic resection of a definite extent of myocardium (5–10 g) through the basal septum below the aortic valve, distally to the point of mitral leaflet–septal contact. The procedure determines an immediate significant reduction of LVOTO and SAM-related mitral regurgitation, thus improving clinical status. Over the years, the original surgical procedure has substantially evolved. Septal resection is now often extended distally to the level of the papillary muscles in order to avoid residual midventricular obstruction. To this end, pre-operative planning with cardiovascular magnetic resonance provides high resolution images of septal morphology which allows a standardized and apically extended septal excision and optimizes results [13]. A novel technique has recently been proposed for patients in whom the obstruction is localized distally at the midventricular level and the transaortic approach does not allow an adequate resection. The technique exploits the application of cryoenergy in order to improve the transaortic exposure of the interventricular septum and enable surgeons to perform very distal myectomies [14]. When concomitant papillary muscle abnormalities are present, dissection and reduction of the anomalous papillary apparatus along with chordal cutting and even plication of the mitral valve are performed as part of the contemporary myectomy operation in order to completely eliminate LVOT obstruction (Fig. 1).

In a tailored fashion, the surgical myectomy can be combined with additional procedures, as required, including mitral valve replacement, posterior-superior realignment of the papillary muscles, partial excision and mobilization of papillary muscles, anterior mitral leaflet plication, anterior leaflet extension, and coronary revascularization. Occasionally, surgical correction of concomitant lesions of the aortic valve and/or fibrous (usually discreet) subaortic stenosis can be performed [15, 16].

In experienced centers, long-term surgical results are excellent. More than 90% of patients who undergo surgery are subsequently asymptomatic and can lead a normal life. Post-operative complications include atrioventricular block, ventricular septal defect, and aortic regurgitation, and have a lower frequency for experienced surgeons. Nevertheless, surgical myectomy causes left bundle branch block in many cases, and therefore, patients with preexistent right bundle branch should be informed of a high risk of requiring a pacemaker post-operatively [17]. ASA may selectively be considered in such patients. In-hospital mortality in centers of excellence has decreased significantly in the last two decades, to contemporary values < 1%. However, reports from the US Nationwide Inpatient Database have revealed that most centers performing surgical myectomies produce very small numbers of procedures (median 1.0 per year) and that low volume was associated with higher mortality, longer length of stay, and higher costs. A fourfold increase in mortality was found in the lowest compared to highest volume tertile, reflecting the importance of an adequate team learning curve in this procedure [18].

## Alcohol Septal Ablation

**Fig. 2:**
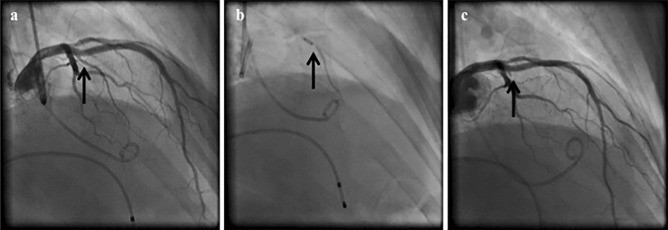
Coronary angiography during alcohol septal ablation. **a** Left coronary angiography shows the target septal branch (*arrow*). **b** Injection of contrast dye through the central lumen of the inflated balloon determines the supply area of the septal branch and excludes leak into the LAD. **c** Occluded septal branch (arrow) after balloon retraction 10 min after last alcohol injection without damage of the left anterior descending artery. ASA, alcohol septal ablation; LAD, left anterior descending. Arrows indicate the target septal branch. ( Reproduced from: Pelliccia F et al. Int J Cardiol. 2020;304:86–92,with permission) [6••]

ASA is a minimally invasive catheter-based technique for achieving septal reduction first reported by Sigwart in 1995 [19]. After its inception, use of the procedure was restricted to inoperable highly symptomatic patients. Since then, despite the absence of large-scale randomized studies, the growing volume of observational data has attracted the interest of clinical and interventional cardiologists, proposing ASA as a first-line intervention in septal reduction therapy as compared with surgery [20]. The procedure consists in the selective infusion of absolute ethanol into a septal artery (most often the first or the second septal perforator branch) supplying the LV side of the basal or mid-cavitary septum (Fig. 2). The rationale is to determine an alcohol-induced occlusion of the perforator branch, with a controlled, limited infarct in the septum [21••]. The “infarcted” tissue progressively turns from hypertrophic “viable” myocardium to thin “akinetic” scar, producing an important reduction in the dynamic LVOTO and SAM of the mitral valve. The evaluation of outflow geometry, septum morphology, and valve apparatus anatomy is crucial in predicting ASA feasibility. The anatomy of coronary circulation is always assessed by coronary angiography, to evaluate vessels’ distribution and detect concomitant atherosclerosis. The existence of anomalies of the LV outflow tract, mitral valve, and papillary muscles (i.e., subaortic membrane, abnormally elongated anterior mitral leaflets, and aberrant papillary muscle insertion) must be excluded. An anterior septal thickness ≥ 17 mm is technically preferable to perform a safe procedure, minimizing the risk of iatrogenic ventricular septal defect [21••].

The long-term results from a multinational ASA registry show that 89% of patients after ASA were in NYHA class 1 or 2, the mean decrease of LVOTO was 76%, and the 30-day mortality rate was 1% [22]. Indeed, perioperative mortality in expert centers is low and largely comparable to surgical myectomy [23]. As for surgery, however, efficacy and safety are strictly related to patient volume and operator expertise. The most prominent complication associated with ASA is the need for a permanent pacemaker due to complete atrioventricular block (around 10% risk in large multicenter observations) [24]. Since the right bundle branch block occurs in more than 50% of cases, the risk of complete atrioventricular block is highest in patients with preexisting left bundle branch block. Other peri-procedural complications have a significantly lower frequency. Noteworthy, late left ventricular dysfunction or a higher rate of sudden cardiac death due to malignant ventricular arrhythmias has not been observed [25].

Long-term benefits include reductions in mitral regurgitation and in LV end-diastolic pressure, reduced left atrial pressure and size, regression of LV hypertrophy, and relief of pulmonary hypertension [23]. Septal ablation leads to improvement in heart failure symptoms and exercise capacity measurable by higher peak oxygen consumption. ASA is less invasive than surgical myectomy, requires less hospitalization, and has a shorter recovery period [2, 21••].

## Myectomy vs Alcohol Septal Ablation

There is no current or completed randomized trial comparing surgical myectomy vs ASA and all comparisons are based on the results of retrospective investigations or meta-analyses of observational studies. Unfortunately, a randomized clinical trial assessing outcome appears unfeasible, based on sample power calculations [26]. Thus, existing recommendations are based on observational findings and expert consensus. The 2014 European Society of Cardiology HCM guidelines does not take a clear stance in favor of either procedure, but simply emphasizes that ASA is controversial in children, adolescents, and young adults for the absence of long-term data on the late effects of a myocardial scar in these groups [4]. Conversely, the 2020 American guidelines for the diagnosis and treatment of HCM state that myectomy should be preferred over ASA [5], but recommend ASA — when feasible and performed in experienced centers — in adult patients with symptomatic obstructive HCM in whom surgery is contraindicated or risk is considered unacceptably high because of serious comorbidities or advanced age.

Both surgical myectomy and ASA may provide substantial symptomatic improvement, increase exercise capacity, and relieve LVOTO [24]. In older adults (≥ 65 years), special considerations need to be made, including the higher risk of concomitant coronary artery disease and atrioventricular block. The benefit of septal reduction therapy in mildly symptomatic patients (NYHA class II), with moderate-to-severe atrial dilation, moderate-to-severe SAM-related mitral regurgitation, or recurrent atrial fibrillation, is still unclear [27]. However, in expert centers with low complication rate and a perioperative mortality now approaching zero, interventions are considered in such patients, as the favorable impact on cardiac remodeling and outcome is likely to be greater than for highly symptomatic patients with structural evidence of disease progression. Notably, patients treated with ASA have shown comparable long-term survival to patients with myectomy [24] and similar functional improvement [28], although the abolition of gradients is not as radical as with surgery. Lastly, the arrhythmic risk profile of patients with HCM does not appear to be affected differently by surgery and ASA. Indeed, most of the studies comparing the two invasive techniques have reported a similar frequency of ventricular arrhythmias and sudden cardiac death [23]. Observational data suggest that surgery can decrease rates of subsequent ventricular arrhythmias [27••]. Similarly, registries and meta-analyses provide reassurance that ASA does not increase the risk of late sudden cardiac death [25], thus disproving initial concerns regarding the arrhythmogenic potential of the scar generated by ASA [23]. Overall, available scientific evidences support the 2014 European Guideline Statement granting equipoise between the selection of myectomy or ASA in the adult patient with drug-refractory HCM [4].

## Role of a Cardiomyopathy Team

In each patient with symptomatic, obstructive HCM, the optimal therapeutic choice should be made on an individual basis with a multifactorial approach. A preliminary assessment of septal anatomy and the mitral valve in order to exclude the need for surgical treatment is mandatory, due to the intrinsic limitation of ASA and the risk of perforation. Likewise, the risk of conduction blocks requiring permanent pacemaker implantation with ASA and surgery should be considered based on the ECG at presentation [4, 5]. ASA is controversial and therefore best avoided in children, adolescents, and very young adults due to lack of long-term data in these groups [2]. Likewise, ASA should be avoided in patients with severe septal hypertrophy exceeding 30 mm. Finally, the final decision regarding the choice of the procedure should take into account patient preferences and local expertise. While high-volume centers should always be preferred for any intervention, the availability of a particularly skilled and experienced surgeon or interventional cardiologist may tilt the balance in favor of myectomy or ASA in a specific regional setting.

Whenever possible, a shared decision-making approach should always be pursued, discussing the risks and benefits of each approach, then understanding the needs and preferences of the individual patient. In order to choose the best-personalized treatment, it is crucial that the entire decision process is carried out with a multidisciplinary approach by a cardiomyopathy team working in dedicated centers of excellence [2].

The concept of “heart team,” a team of clinical or non-invasive cardiologists, cardiac surgeons, and interventional cardiologists, has shown in recent years to improve the decision between angioplasty and surgery in multivessel coronary artery disease or between percutaneous and surgical to face the decision-making of aortic stenosis [29]. Similarly, in the case of HCM, a “Cardiomyopathy Team” should analyze and interpret diagnostic evidence, put into context the clinical condition of the patient, and determine the need—or otherwise—for an intervention and the likelihood of safe and effective septal reduction with either ASA or myectomy. This team should be composed by at least one clinical cardiologist, an interventional cardiologist, and a cardiac surgeon with a recognized experience in the management of HCM, discussing benefits and risks of different strategies [6••]. At present, HCM centers with high-volume surgical programs performing septal myectomy are not universally available to all patients who are candidates for and require septal reduction therapy. Moreover, procedural volumes are still low in most hospitals performing these procedures, and in-hospital mortality is significantly higher when patients undergo either surgical myectomy or alcohol ablation at low-volume institutions. Although specific data are lacking, a minimum of 10 ASA or 10 septal myectomies per operator per year seems to be a reasonable caseload to be required in order to maintain competence in the field [4].

## Future Perspectives

In summary, surgical myectomy and ASA are both effective options for management of LVOTO in HCM patients whose symptoms are refractory to pharmacological treatment. Optimizing efficacy while reducing pacemaker dependency and improving long-term survival is the ultimate goal of both procedures. In the absence of randomized trials comparing the two methods, the best treatment for each individual patient should be determined by a “Cardiomyopathy Team” with expertise in both procedures (Table 1). The choice should take into consideration anatomical and functional features as well as concomitant cardiac and non-cardiac morbidities. Final success and complication rates depend heavily on the experience of the surgeon or interventional cardiologist involved. Patients’ preferences, following a detailed discussion of the available options, play an increasingly important role in the decision-making process [30].

**Table 1:** Clinical, morphologic, and procedural criteria influencing decision process of optimal individual septal reduction therapy

**Criteria**	**Myectomy**	**Alcohol septal ablation**
**Patients age**	• Pediatric • Adolescent • Adults	• Adults • High surgical risk patients
**Site of obstruction**	• Basal • Midventricular • Apical	• Basal
**MV insufficiency**	• Secondary to SAM • MV disease or abnormal papillary m	• Secondary to SAM
**LV hypertrophy**	• Any magnitude of hypertrophy	• Basal hypertrophy (range 17–30 mm)
**Coronary artery**	• Any congenital anomaly • Multiple vessel disease	• Suitable first or second septal artery • 1 (–2) vessel disease
**Other associated cardiac conditions**	• Extensive endocardial LVOT fibrosis • Aortic valve disease • Aortic dilation	
**Complexity of procedure**	• Surgery with extracorporeal circulation	• Less invasive
**Post procedure risk for pacemaker dependency**	• 2–4% after surgery • Up to 50% with preexisting RBBB	• 10 to 20% after procedure • Up to 50% with preexisting LBBB
**Hemodynamic success**	• Immediate	• Delayed (3–12 months)
**Redo**	• Less than 1%	• 7–20%
**Post procedure length of hospital stay**	• 6–9 days ± rehabilitation	• 3–4 days

*SAM* systolic anterior motion of the mitral valve, *HLM* heart lung machine, *RBBB* right bundle branch block, *LBBB* left bundle branch block
